# Further evidence for the genetic association between *CACNA1I* and schizophrenia

**DOI:** 10.1186/s41065-017-0054-0

**Published:** 2018-01-02

**Authors:** Yijun Xie, Di Huang, Li Wei, Xiong-Jian Luo

**Affiliations:** 1 Clinical Laboratory, Sichuan Academy of medical sciences & Sichuan provincial people’s hospital, Chengdu, 610072 China; 2 Key Laboratory of Animal Models and Human Disease Mechanisms of the Chinese Academy of Sciences & Yunnan Province, Kunming Institute of Zoology, Chinese Academy of Sciences, Kunming, Yunnan 650223 China; 3 Clinical Laboratory, The fourth people’s hospital of Chengdu, Province, Chengdu, Sichuan 610000 China

**Keywords:** Schizophrenia, *CACNA1I*, Genetic association, Gene expression

## Abstract

**Background:**

Recent large-scale genome-wide association studies (GWAS) have showed that the neuronal calcium signaling has pivotal roles in schizophrenia (SCZ) in populations of European of ancestry. However, it is not known if calcium signaling pathway genes are also associated with SCZ in Han Chinese population.

**Methods:**

Here we investigated the association between genetic variants in three calcium signaling pathway genes (*CACNB2*, *CACNA1C* and *CACNA1I*) and SCZ in 1615 SCZ cases and 1597 controls.

**Results:**

A single nucleotide polymorphism (SNP) (rs4522708) in *CACNA1I* is significantly associated with SCZ in our Chinese sample (OR_A allele_ = 1.19, corrected *P* = 0.042), suggesting that *CACNA1I* may also be a risk gene for SCZ in Chinese population. Of note, the risk allele (A allele) of SNP rs4522708 is same in European and Chinese populations. Meta-analysis of Chinese and European samples further strengthened the association of rs4522708 with SCZ (OR_A allele_ = 1.074, *P* = 6.26 × 10^−11^). Expression analysis showed that *CACNA1I* was significantly up-regulated in hippocampus of SCZ cases compared with controls, implying that dysregulation of *CACNA1I* may have a role in schizophrenia pathogenesis.

**Conclusions:**

Our study suggests that *CACNA1I* is a risk gene for SCZ in Chinese population and provides further evidence that supports the potential role of neuronal calcium signaling in schizophrenia.

## Background

Schizophrenia (SCZ) is a severe mental disorder that affects about 1% of the world’s population [1]. The core symptoms of SCZ include delusions, hallucinations and cognitive dysfunction [2, 3]. Adoption, family and twin studies indicated that SCZ has a strong genetic component [4]. The heritability of SCZ was estimated about 80% [4, 5], suggesting that genetic factor plays a crucial role in SCZ. Though numerous genetic linkage and association studies have been carried out in different ethnic populations and multiple susceptibility genes have been identified [6–8], only limited risk genes have been consistently replicated in diverse populations. The advent of genome-wide association studies (GWAS) provides an opportunity to dissect the genetic basis of SCZ. In the past decade, multiple GWAS have been performed in world’s populations and multiple promising risk variants and genes have been reported [9–13]. In 2014, the schizophrenia working group of the psychiatric genomics consortium (PGC2) conducted a large-scale meta-analysis of GWAS through using 36,989 SCZ cases and 113,075 controls [13]. Over 100 SCZ-associated loci have been identified by PGC. A detailed characterization of the risk loci showed that calcium signaling pathway genes may play pivotal roles in SCZ [13]. Two calcium signaling pathway genes (including *CACNA1C* and *CACNA1I*) showed robust association with SCZ in PGC2, strongly implying these genes may be involved in SCZ pathogenesis. In addition, another calcium signaling pathway gene (*CACNB2*) was also reported to be associated with SCZ [14]. A recent trans-ancestry meta-analyses of Chinese and PGC2 samples (a total of 43,175 cases and 65,166 controls) further supports the association between *CACNB2, CACNA1C, CACNA1I* genes and schizophrenia [15]. These findings strongly suggest that neuronal calcium signaling pathway is involved in schizophrenia. To further investigate if these calcium signaling pathway genes are also associated with SCZ in Han Chinese population, we performed a replication study through recruiting 1615 SCZ cases and 1597 controls. We first genotyped three SNPs (one SNP in *CACNB2*, one SNP in *CACNA1C* and one SNP in *CACNA1I*) and tested their associations with SCZ in a Chinese sample. We then explored the expression level of these three genes in schizophrenia cases and controls. Our study indicates that a SNP in *CACNA1I* is significantly associated with SCZ in Chinese population, suggesting that *CACNA1I* is also a risk gene for SCZ in Han Chinese.

## Materials and methods

### Study subjects

We included 1615 SCZ cases and 1597 controls in this study. All of SCZ cases were from local mental health hospitals (or centers) and some of the patients have been reported in our previously studies [16, 17]. Diagnosis was finished by at least two experienced psychiatrists with DSM IV criteria. 61.3% of the SCZ cases were females and the ages of the SCZ cases range from 13 to 81 years (35.1 ± 13.26 years). Patients with history of head injuries and drug abuse were excluded in this study. Healthy controls were local residents and screened for lifetime history of mental illness. 63.9% of the controls were females and the ages of the controls range from 17 to 74 years (38.08 ± 12.31 years). Written informed consents were obtained from all the studied subjects and this the research was approved by the internal review board of Kunming Institute of Zoology, CAS. More detailed information about the sample description can be found in our previous studies [16, 17].

### SNP selection

As the goal of this study is to explore if the calcium signaling pathway genes reported in European populations are also associated with SCZ in Han Chinese, we selected three previously reported risk genes for SCZ, including *CACNB2* [14], *CACNA1C* and *CACNA1I* [13]. All of these three genes encode voltage-dependent calcium channel subunits. *CACNB2* encodes voltage-dependent L-type calcium channel subunit beta-2. *CACNA1C* encodes Ca_v_1.2, an alpha-1 subunit of a voltage-dependent calcium channel. And *CACNA1I* encodes Ca_v_3.3, an alpha-1I subunit of a voltage-dependent calcium channel. For *CACNB2*, we selected SNP rs4748478, which showed significant association with SCZ in PGC2 (*P* = 8.0 × 10^−3^). For *CACNA1C*, we selected SNP rs1006737, which frequently reported to be associated with SCZ in previously studies [18–22]. For *CACNA1I*, we selected SNP rs4522708, which is also significantly associated with SCZ in PGC2. Of note, rs1006737 and rs4522708 reached genome-wide significance level in PGC2 (*P* = 1.09 × 10^−16^ and *P* = 2.41 × 10^−16^, respectively) [13].

### Genotyping

SNP genotyping was performed using SNaPShot method as described previously [16]. Single nucleotide extension strategy was used for genotyping. Briefly, DNA fragments containing the SNP site were amplified first using PCR. The PCR products were then treated with Shrimp Alkaline Phosphatase (SAP) and Exonuclease I (ExoI), which remove primers and unincorporated dNTPs. The cleaned PCR products were then used as templates for single nucleotide extension. The genotyping primers were designed to stop just one base upstream of the interest SNP. As ddNTPs were used, so the polymerase extends the primer by one nucleotide, adding a single ddNTP to its 3′ end. ABI 3730 was used to determine which base was added. More detailed information about SNaPShot genotyping method can be found in our previous study [16].

### Statistical analysis

We used PLINK (v1.9) [23] to perform the association test (chi-square allelic test with 1 df). Bonferroni correction was used to correct the *P* values. Meta-analysis was performed using metafor package (http://www.metafor-project.org/) [24] implemented in R as described previously [25] and Fixed-effect model was used.

### Expression analysis of CACNA1I in SCZ cases and controls

To explore if expression of these *CACNA1I* was changed in SCZ cases compared with controls, we examined the expression level of *CACNA1I* in SCZ subjects and controls using GES53987 [26]. In brief, 19 SCZ cases and 19 controls were included in GSE53987 and gene expression in hippocampus was measured. The raw expression values were processed as described in the original study [26] and *student t* test was used to determine if expression of *CACNA1I* is significantly changed in SCZ cases compared with controls.

### Linkage disequilibrium analysis

Linkage disequilibrium (LD) analysis were performed using genotype information from the 1000 genomes project [27]. As all of the subjects included in this study were Han Chinese, we only used the genotype information of 103 Chinese subjects (CHB) from the 1000 genomes project to construct and calculate LD values (r [2]) among the SNPs. Haploview program was used to calculated LD [28].

### Expression analysis of CACNA1I in human tissues

To explore the expression of *CACNA1I* in human tissues, we downloaded the RNA-seq-based expression data from the study of Fagerberg et al. [29] Briefly, expression data from 27 different tissues of 95 human individuals were used.

## Results

### A SNP in *CACNA1I* is significantly associated with SCZ in Chinese population

We successfully genotyped the three selected SNPs (rs4748478, rs1006737 and rs4522708) in most of our samples. The overall genotyping call rate of these three SNPs exceeded 0.99. Hardy-Weinberg equilibrium analysis showed that all of the three SNPs are in Hardy-Weinberg equilibrium. Single SNP association test indicated that rs4748478 and rs1006737 were not associated with SCZ in our sample (*P* > 0.05). However, we found that rs4522708 is significantly associated with SCZ (reference allele: A, OR_A allele_ = 1.19, *P* = 0.0145) (Table 1). Considering that we tested three SNPs in this study, we corrected *P* values using Bonferroni correction. Again, we found rs4522708 still showed significant association with SCZ even Bonferroni correction was applied (*P* = 0.0435), suggesting this SNP is also associated with SCZ in Chinese population.

**Table 1:** Association significance between the studied SNPs and schizophrenia

SNP id	Chr	A1^a^	A2^b^	Freq_A^c^	Freq_U^d^	χ^2e^	OR^g^	P	P_corrected_^f^
rs4748478	10	G	A	49.5%	48.8%	0.299	1.028	0.585	1.0
rs1006737	12	A	G	5.43%	4.96%	0.725	1.102	0.395	1.0
**rs4522708**	**22**	**A**	**G**	**86.11%**	**83.92%**	**5.981**	**1.188**	**0.0145**	**0.0435**

^a^Reference allele. ^b^Alternative allele. ^c^Frequency of reference allele in cases, ^d^Frequency of reference allele in controls. ^e^Chi square value. ^f^P was corrected by Bonferroni correction. ^g^Odds ratio is based on reference allele. Significant associations were shown in bold

SNP rs4522708 has two alleles, A and G. The risk allele of rs4522708 in our sample is A allele (OR_A allele_ = 1.19, *P* = 0.0145). Of note, we found the risk allele of rs4522708 is also the A allele in PGC2 (OR_A allele_ = 1.071, *P* = 2.41 × 10^−10^) (35,476 schizophrenia cases and 46,839 controls). We thus performed a meta-analysis through combing our samples (1615 cases and 1597 controls) with samples from PGC2 (35,476 schizophrenia cases and 46,839 controls). A total of 37,091 SCZ cases and 48,436 controls were included in the meta-analysis. Heterogeneity test showed there was no heterogeneity in the combined samples (*P* > 0.05). The meta-analysis (fixed-effect model) further strengthened the association between rs4522708 and SCZ (OR_A allele_ = 1.074, two-tailed *P* = 6.26 × 10^−11^). Taken together, these results suggest that rs4522708 may represent an authentic risk variant for SCZ.

### *CACNA1I* was significantly up-regulated in SCZ cases compared with controls

**Fig. 1:**
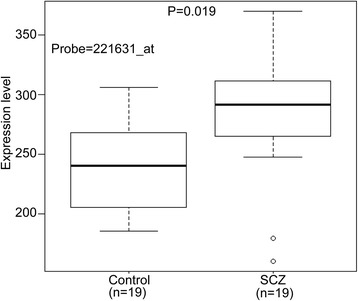
*CACNAI1* was significantly up-regulated in hippocampus of schizophrenia cases compared with controls. Nineteen schizophrenia cases and 19 controls were included in analysis

Our genetic association results indicate that *CACNA1I* is also associated with SCZ in Chinese population. To further explore the potential role of *CACNA1I* in schizophrenia pathogenesis, we examined *CACNA1I* expression in hippocampus of SCZ cases and controls. We found that *CACNA1I* was significantly up-regulated in hippocampus of SCZ cases compared with controls (*P* = 0.019, Fig. 1), suggesting dysregulation of *CACNA1I* in schizophrenia cases. This expression analysis provided further evidence that supports the involvement of *CACNA1I* in schizophrenia.

### *CACNA1I* was highly expressed in human brains compared with other tissues

**Fig. 2:**
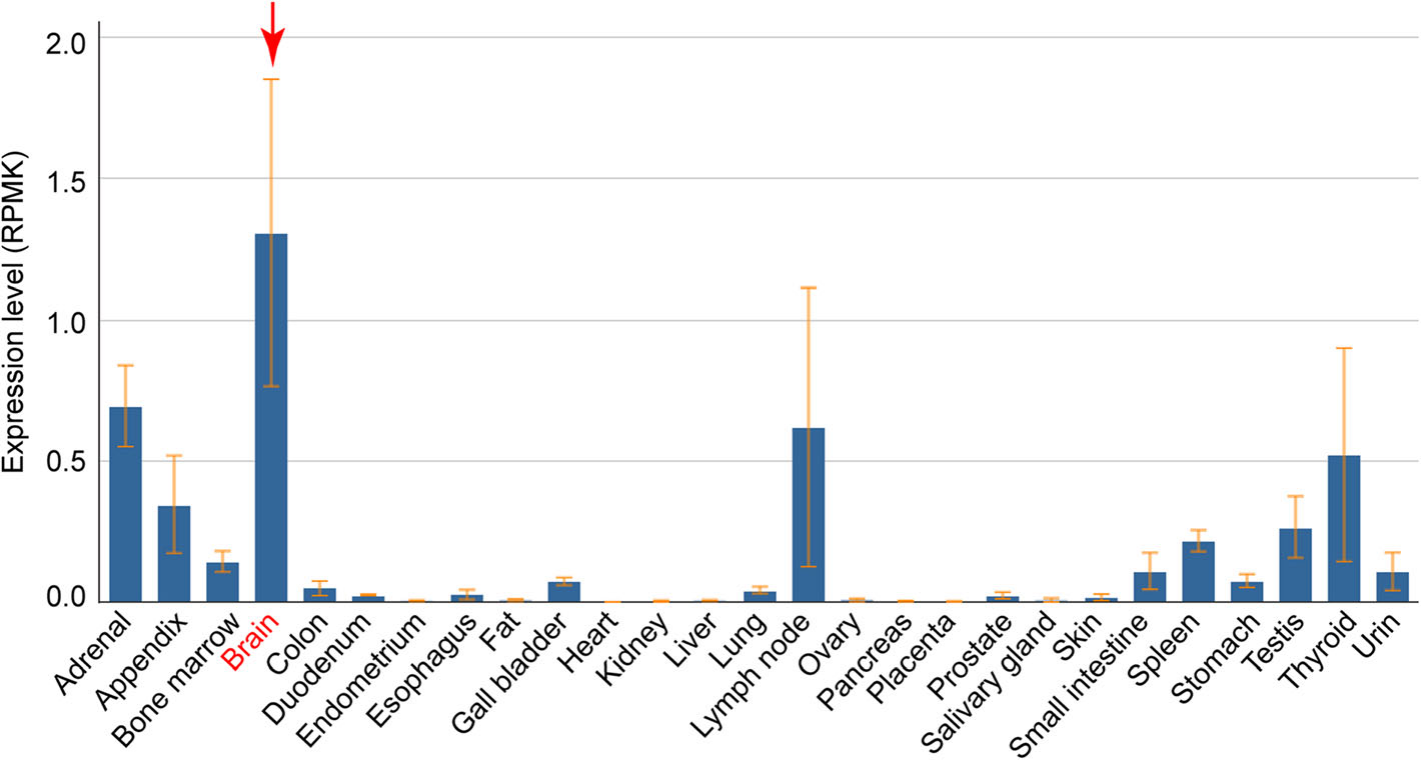
*CACNAI1* is highly expressed in human brain tissues compared with other tissues. The arrowhead shows the human brain tissue

We found that *CACNA1I* was highly expressed in human brain tissues compared with other tissues (Fig. 2). In fact, *CACNA1I* has the highest expression level in human brains, suggesting this gene may have a role in human brain.

## Discussion

Recent large-scale GWAS of schizophrenia (PGC2) have frequently reported that calcium signaling pathway genes were associated with schizophrenia. Of note, genetic variants in *CACNA1C* and *CACNA1I* reached genome-wide significance level in PGC2. In addition, previous study also showed that *CACNB2* gene was a risk gene for SCZ. Though the associations between these three calcium signaling pathway genes and SCZ have been frequently reported, we noticed that most of the studies were from populations of European ancestry. It is not known if these three calcium signaling pathway genes were also associated with SCZ in Han Chinese. In this study, we genotyped three SNPs in *CACNB2, CACNA1C* and *CACNA1I* in 1615 SCZ cases and 1597 controls (All of the subjects were Han Chinese). We found that a SNP in *CACNA1I* is significantly associated with SCZ in our Chinese sample, suggesting *CACNA1I* may also represent a risk gene for SCZ in Chinese population. To further explore the potential role of *CACNA1I* in SCZ, we examined expression analysis and found that expression of *CACNA1I* was significantly up-regulated in SCZ cases compared with controls, implying dysregulation of *CACNA1I* may have a role in SCZ.

**Fig. 3:**
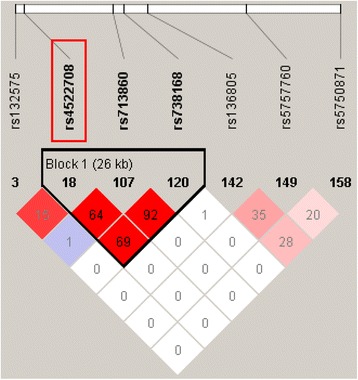
Linkage disequilibrium analysis among the SNP investigated in this study (rs4522708) and the SNPs that were significantly associated with schizophrenia in previous study. The significant SNP observed in this study (rs4522708) is marked with red box. The values of linkage disequilibrium (r [2]) were shown in the box

Of note, a recent study also showed that genetic variants in *CANCA1I* were significantly associated with SCZ in Uighur Chinese population [30]. Six SNPs (rs132575, rs713860, rs738168, rs136805, rs5757760 and rs5750871) were found to be associated with SCZ in the study of Xu et al. [30] We studied the linkage disequilibrium between these six SNPs and the SNP investigated in our study (i.e., rs4522708). We found that rs4522708 is linked with two previous reported SNPs (rs713860 and rs738168) (Fig. 3). However, rs4522708 is not linked with other significant SNPs reported in previous study [30]. These LD analysis results suggested that several independent genetic variants in *CACNAI1* were associated with SCZ. The observation of significant association between *CACNA1I* and SCZ in two independent Chinese samples implies that *CACNAI1* may represent an authentic risk gene for SCZ. Consistent with these findings, a recent trans-ancestry meta-analyses of Chinese and PGC2 samples (a total of 43,175 cases and 65,166 controls) provided further evidence for the association between *CACNB2, CACNA1C, CACNA1I* genes and schizophrenia [15]. These results strongly suggest that neuronal calcium signaling pathway plays a pivotal role in schizophrenia.

There were several limitations of this study. First, only one SNP was selected for each of the three calcium signaling pathway genes in this study. Thus, the SNP coverage is limited. We could not exclude if other SNPs in *CACNB2* and *CACNA1C* were associated with SCZ. Second, the sample size is relatively small in this study. Further replication study with larger sample size is needed to validate our results. Taken together, our study showed that *CACNA1I* is also associated with SCZ in Chinese population. Our results provide further evidence that support the association of *CACNA1I* with SCZ.
